# *Glycyrrhiza glabra* HPLC fractions: identification of Aldehydo Isoophiopogonone and Liquirtigenin having activity against multidrug resistant bacteria

**DOI:** 10.1186/s12906-018-2207-8

**Published:** 2018-05-02

**Authors:** Hazir Rahman, Ilyas Khan, Anwar Hussain, Abdelaaty Abdelaziz Shahat, Abdul Tawab, Muhammad Qasim, Muhammad Adnan, Mansour S. Al-Said, Riaz Ullah, Shahid Niaz Khan

**Affiliations:** 1 Department of Microbiology, Abdul Wali Khan University Mardan, Mardan, Pakistan; 2 Department of Microbiology, Kohat University of Science & Technology, Kohat, Pakistan; 3 Department of Botany, Abdul Wali Khan University, Mardan, Pakistan; 4 Department of Pharmacognosy and Medicinal Aromatic, and Poisonous Plants Research Center, College of Pharmacy King Saud University, Riyadh, 11451 Saudi Arabia; 5 Phytochemistry Department, National Research Centre, P.O. Box 12622, 33 El Bohouth st., Dokki, Giza, Egypt; 6 National Institute for Biotechnology and Genetic Engineering, Faisalabad, Pakistan; 7 Department of Botany, Kohat University of Science & Technology, Kohat, Pakistan; 8 Department of Zoology, Kohat University of Science & Technology, Kohat, Pakistan

**Keywords:** *Glycyrrhiza glabra*, HPLC fractionation, Anti-MDR activity, Ellagic acid 6-aldehydo-isoophiopogonone, Liquirtigenin

## Abstract

**Background:**

Medicinal plants have been founded as traditional herbal medicine worldwide. Most of the plant’s therapeutic properties are due to the presence of secondary metabolites such as alkaloids, glycosides, tannins and volatile oil.

**Methods:**

The present investigation analyzed the High-Pressure Liquid Chromatography (HPLC) fractions of *Glycyrrhiza glabra* (Aqueous, Chloroform, Ethanol and Hexane) against multidrug resistant human bacterial pathogens (*Escherichia coli, Acinetobacter baumannii, Staphylococcus aureus* and *Pseudomonas aeruginosa*). All the fractions showed antibacterial activity, were subjected to LC MS/MS analysis for identification of bioactive compounds.

**Results:**

Among total HPLC fractions of *G. glabra* (*n* = 20), three HPLC fractions showed potential activity against multidrug resistant (MDR) bacterial isolates. Fraction 1 (F1) of aqueous extracts, showed activity against *A. baumannii* (15 ± 0.5 mm)*.* F4 from hexane extract of *G. glabra* showed activity against *S. aureus* (10 ± 0.2 mm)*.* However, F2 from ethanol extract exhibited activity against *S. aureus* (10 ± 0.3 mm)*.* These active fractions were further processed by LC MS/MS analysis for the identification of compounds. Ellagic acid was identified in the F1 of aqueous extract while 6-aldehydo-isoophiopogonone was present in F4 of hexane extract. Similarly, Liquirtigenin was identified in F2 of ethanol.

**Conclusions:**

*Glycyrrhiza glabra* extracts HPLC fractions showed anti-MDR activity. Three bioactive compounds were identified in the study. 6-aldehydo-isoophiopogonone and Liquirtigenin were for the first time reported in *G. glabra*. Further characterization of the identified compounds will be helpful for possible therapeutic uses against infectious diseases caused by multidrug resistant bacteria.

## Background

Medicinal plants are used for the treatment of various infections [1, 2]. These plants contributed as a source of inspiration for novel therapeutic compounds [3]. The medicinal value of plants is due to the presence of a wide variety of secondary metabolites including alkaloids, glycosides, tannins, volatile oil and terpenoids [4]. The distribution of *G. glabra* plant is worldwide, the plant is reported from different regions e.g. Central Asia, Spain, Italy, China, Turkey, Iran, India [5] and Pakistan.

*Glycyrrhiza glabra* has a sweet wood and usually employed medicinally as an expectorant and carminative. Moreover, since long time *G. glabra* is used as a potential therapeutic herb found in various parts of the world. The herb is also used for the treatment of resistant microbes involved in the infections of skin, respiratory tract, urinary system and as anti-ulcer activity. The use of *G. glabra* is also reported in Unani an Ayurveda medicines [6, 7]. Besides, Oil from *G. glabra* is used as a flavoring agent and natural sweetener. The compounds which add these features are glycyrrhizin and some volatile compounds, saponins and flavonoids. Due to these characteristic *G. glabra* oil extract is also used in confectioneries, personal care products, food items, beverages and cosmetics [8]. Phenolics compounds from the root of *G. glabra* are isolated which protect low density lipoproteins and from oxidative damage [9].

Literature data is available on the antibacterial activity of *G. glabra* crude extract; however, no data exist on the biological activity of HPLC fractions from *G. glabra* extracts. In the present study, gradient HPLC fractions were collected, and only these fractions with antibacterial activity were further investigated by LC MS/MS analysis for the identification of bioactive compounds. Findings of the study will be helpful for elucidation of lead molecules for possible therapeutic intervention.

## Methods

The current study was performed to check the antibacterial activity of *G. glabra*’s chromatographic fractions against multidrug resistant (MDR) pathogenic bacterial isolates at the Department of Microbiology, Kohat University of Science and Technology (KUST), Kohat. Ethical approval was waived by the Departmental Review Board (DRB) with a reference number MIC/KUST/2118.

### Processing of *G. glabra*

*G. glabra* (Mulaithi) plant was collected from Khyber Pakhtunkhwa (KPK), Pakistan, and was morphologically identified by plant taxonomist Prof. Dr. Waheed Murad at the Herbarium of Botany department, Kohat University of Science & Technology, where the voucher sample (10,052/GG) was deposited. After identification, *G. glabra* was washed, air dried and chopped. The plant pieces were dried and mashed into powder form for further processing [10]. The powdered plant was subjected to extraction process as described earlier [11].

### Gradient HPLC fractionation

Solidified extracts of plants were processed for HPLC fractionation by dissolving in 60% methanol as described [12].

### Collection of multi drug resistant (MDR) bacteria

Pure cultures of the MDR human were obtained from the Department of Microbiology, KUST and confirmed on the basis of culture, microscopy and biochemical characteristics [13].

### Antibacterial activity of plant extracts

Bacterial cultures were inoculated on Muller Hinton agar. Three HPLC fractions of *G. glabra* plant were poured in three wells of each plate while DMSO was placed as a negative control. Results were interpreted by using standard guidelines [14].

### LC MS/MS analysis

Those fractions which showed antibacterial activity were processed bioactive compounds identification using LC MS/MS (LTQ XL, Thermo Electron Corporation, USA) method as described earlier [15].

### Bioinformatics and data analysis

Structure parameters for each compound were obtained by using online database software (www.chemspider.com).

## Results

A total twenty (*n* = 20) gradient HPLC fractions (Five fraction for each extract) were collected from extracts of *G. glabra* plant and processed for antibacterial activity. The zones of inhibition showed by different fractions of *G. glabra* extracts were measured against known MDR bacterial isolates. Among total twenty HPLC fractions, three fractions of aqueous, hexane and ethanol extract exhibited activity against MDR bacteria.

Among the total HPLC fractions of *G. glabra*, three fractions showed activity against multidrug resistant (MDR) bacterial isolates. When five HPLC fractions from aqueous extracts were checked, only fraction 1 (F1) exhibited a zone of inhibition (15 ± 0.5 mm) against *A. baumannii* (Table 1)*.*

**Table 1:** Zone of inhibition showed by HPLC fractions of *G. glabra*

S.No	Bacteria	Zone of inhibition (mm) by *G. glabra* extracts			
		Aqueous extract	Hexane extract	Ethanol extract	Negative control (DMSO)
		F4	F4	F1	
1	*E. coli*	0	0	0	0
2	*S. aureus*	0	10 ± 0.2	10 ± 0.3	0
3	*A. baumannii*	15 ± 0.5	0	0	0
4	*P. aeruginosa*	0	0	0	0

*F* Fraction, *±* standard error of given value, *DMSO* Dimethyl sulfoxide as negative control

A zone of inhibition (10 ± 0.2 mm) was observed against *S. aureus* (*MRSA*) by fraction 4 (F4) *of* hexan extract of *G. glabra*. When F2 of ethanol extract was processed for antimicrobial activity, a zone of inhibition (10 ± 0.5 mm) was observed against *S. aureus.* All the active fractions were subjected to mass spectrometric analysis for compounds identification (Table 1).

**Fig. 1:**
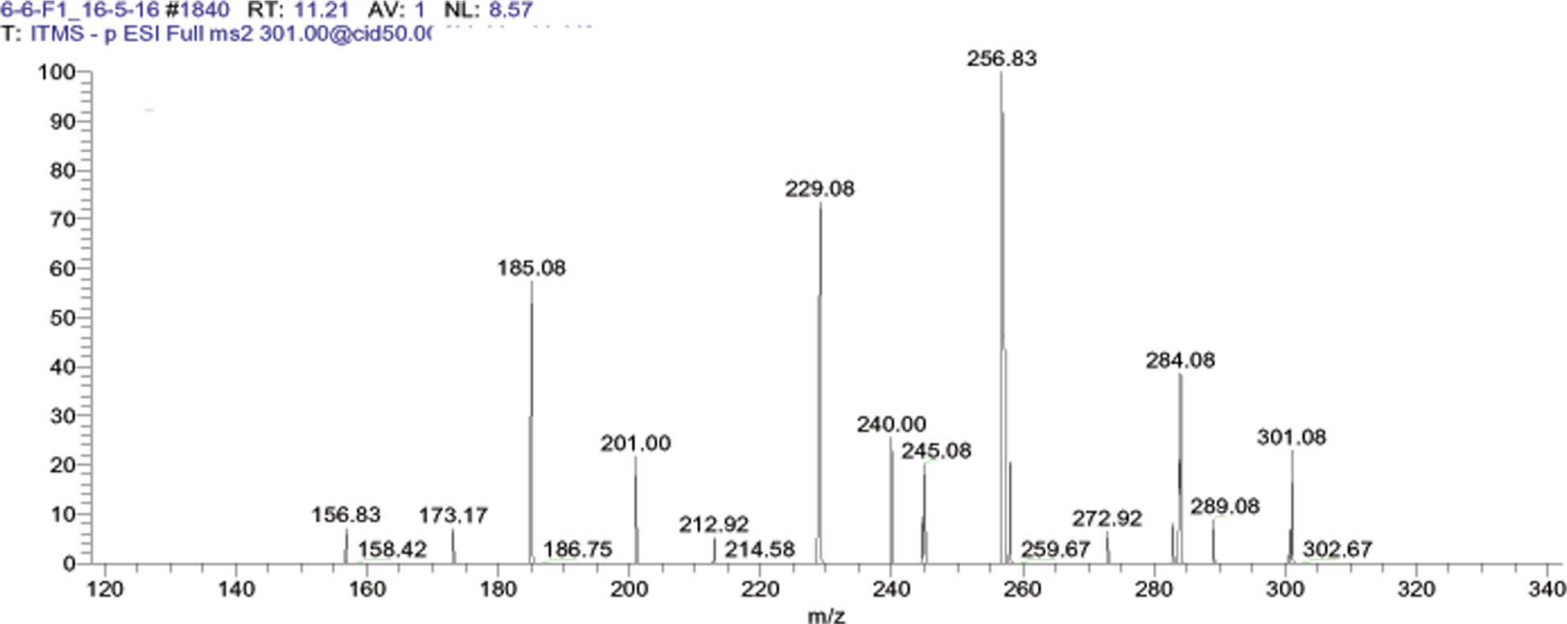
LC MS/MS Chromatogram of fraction 1 (F1) from aqueous extracts of *Glycyrrhiza glabra* showing Ellagic Acid

**Fig. 2:**
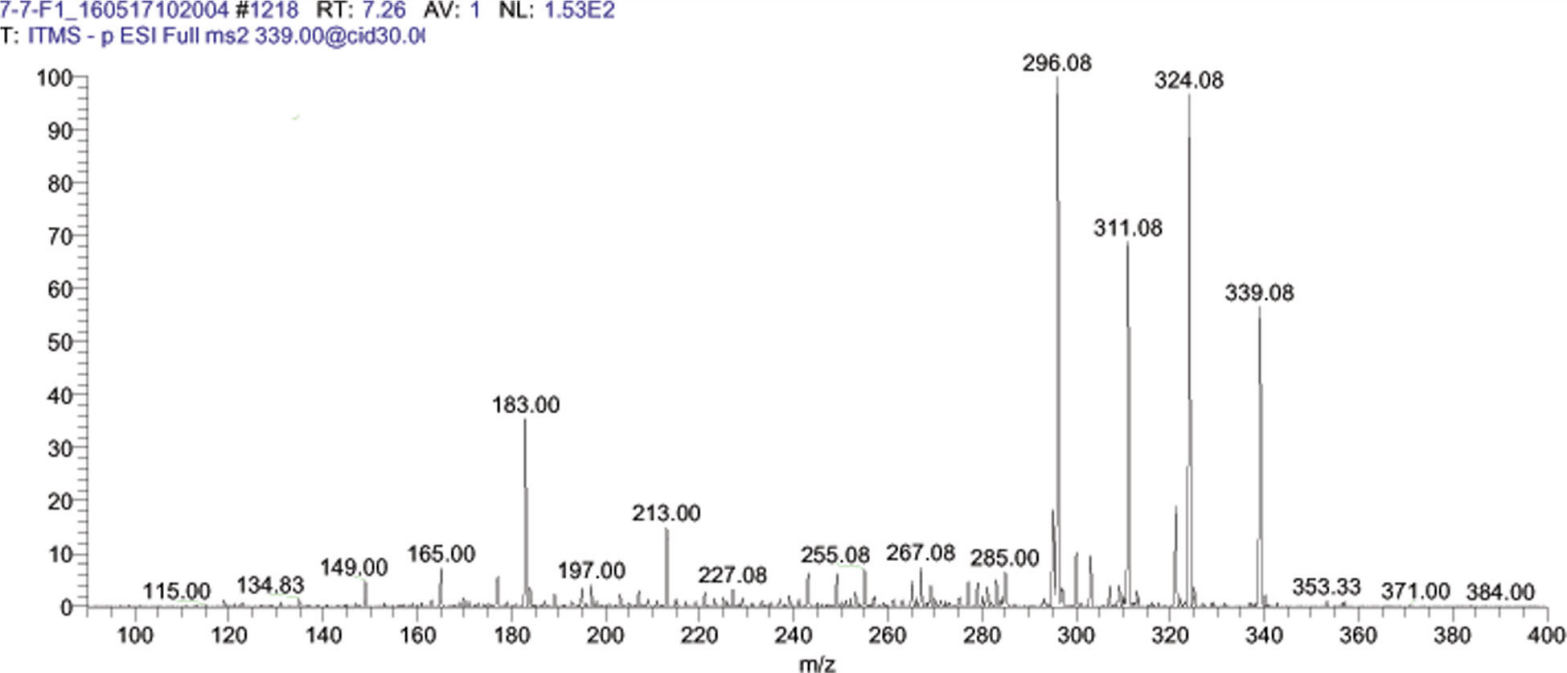
LC MS/MS Chromatogram of fraction 4 (F4) from hexane extracts of *Glycyrrhiza glabra* showing 6-aldehydo-isoophiopogonone

**Fig. 3:**
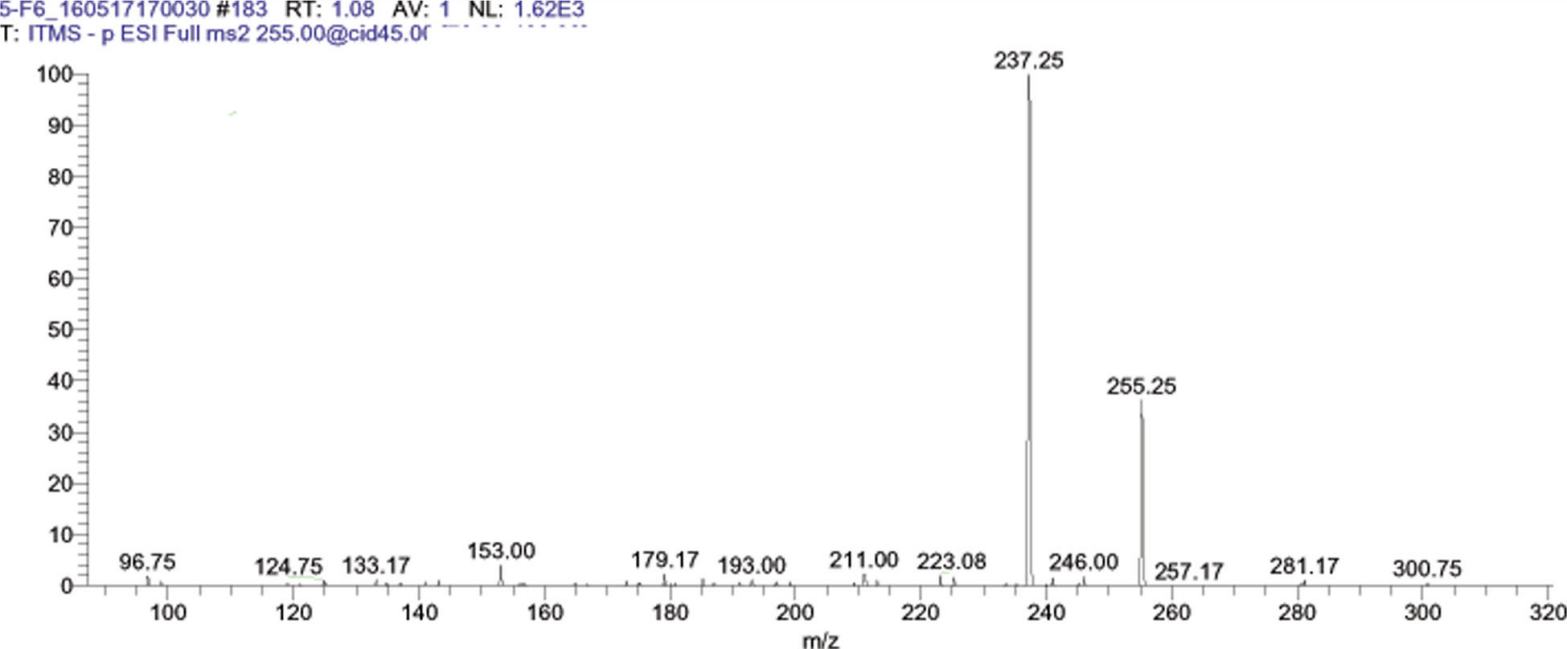
LC MS/MS Chromatogram of fraction 2 (F2) from ethanol extracts of *Glycyrrhiza glabra* showing Liquirtigenin

After LC MS/MS and bioinformatics analysis (Table 2), Ellagic acid was identified in the F1 of aqueous extract of *G. glabra* (Fig. 1). In the F4 of hexane extract, 6-aldehydo-isoophiopogonone was identified (Fig. 2). Lastly Liquirtigenin was identified in the F2 of ethanol extract (Fig. 3).

**Table 2:** Profile of bioactive compounds of *Glycyrrhiza glabra* identified by LC MS/MS analysis

S. No	Fraction source	Fraction ID	Compound Name	Molecular Formula	Molecular Weight (Da)	Structure
1.	Aqueous	F1	Ellagic Acid	C_14_H_6_O	302.193	
2.	Hexane	F4	6-aldehydo-isoophiopogonone.	C_19_H_14_O_7_	354.3	
3.	Ethanol	F2	Liquirtigenin	C_15_H_12_O_4_	256.253

## Discussion

In the present study *G. glabra* was selected for HPLC fractionation and to check the antibacterial activity of these fractions. These plant HPLC fractions were screened against selected multi-drug resistant bacteria. Fraction of chloroform extracts showed considerable activity against *A. baumannii* and *E. coli*. Previously the compounds responsible for their antimicrobial activity were identified by HPLC fractionating plant extract and determining the antimicrobial activity of each fraction against *A. baumannii* [16]. Earlier study has been conducted to check the antimicrobial activity of *G. glabra* extracts prepared in different solvents. In a study the chloroform and acetone extracts of *G. glabra* showed good activity against two Gram positive and two Gram negative bacteria [17].

Among ethanol extract HPLC fractions, only fraction 2 (F2) have antibacterial effect against *S. aureus*. Potential antimicrobial activity of *G. glabra* extracts was reported against *S. aureus* and *E. coli* respectively [18, 19]. Aparajita Gupta (2013) also evaluated the promising activity of methanol and acetone extract of *G. glabra* against *E. coli* and *S. aureus* [20]. The F4 fraction of hexane extract showed considerable antibacterial activity. In a study six medicinal plants including *G. glabra* were investigated for pharmaceuticals and their role in antimicrobial activity against antibiotic resistant bacteria isolated from pharmaceuticals and hospital [21]. Likewise F1 of aqueous showed anti-MDR activity. The above results of a study suggested that the ethanol fraction contained maximum soluble bioactive compounds which may be responsible for the highest antibacterial activity.

Fractions exhibited potential antimicrobial activity was further subjected to LC MS/MS analysis for the identification of bioactive compounds. Fraction 2 from ethanol was identified as Liquirtigenin. 6-aldehydo-isoophiopogonone was identified in fraction 4 of Hexane extract. There is no data reported on 6-aldehydo-isoophiopogonone from *G. glabra*; however, this compound was isolated from *Ophiopogon japonicas* plant as homoisoflavonoids which showed antioxidant activities [22]. Ellagic Acid was identified in fraction 4 of hexane extract. This compound is for the first time reported in *G. glabra*; however other studies reported this compound for its anti-mutagenic, antimicrobial, antioxidant properties [23].

## Conclusions

Among the total HPLC fractions of plant *G. glabra*, three fractions showed activity against multidrug resistant (MDR) bacterial isolates. 6-aldehydo-isoophiopogonone and Liquirtigenin were for the first time reported in *G. glabra*. Further studies on these compounds may lead to the development of new potential antibacterial compounds.

## Abbreviations

DMSO: Dimethyle sulfoxide
HPLC: High performance liquid chromatography
MDR: Multidrug resistant

## References

[1] Mahesh B, Satish S (2008). Antimicrobial activity of some important medicinal plant against plant and human pathogens. World J Agric Sci.

[2] Van-Wyk BE (2002). A review of ethnobotanical research in southern Africa. S Afr J Bot.

[3] Bhojane P, Damle S, Thite A, Dabholkar V (2014). Anti-microbial effects of some leafy vegetables - a comparative analysis. Int Res J Biol Sci.

[4] Talib WH, Mahasneh AM (2010). Antimicrobial, cytotoxicity and phytochemical screening of Jordanian plants used in traditional medicine. Molecules.

[5] Hayashi H, Hattori S, Inoue K, Khodzhimatov O, Ashurmetov O, Ito M, Honda G (2003). Field survey of Glycyrrhiza plants in Central Asia (3). Chemical characterization of G. Glabra collected in Uzbekistan. Chem Pharm Bull.

[6] Lakshmi T, Geetha RV (2011). *Glycyrrhiza Glabra* Linn commonly known as licorice: a therapeutic review. Int J Pharm Pharm Sci.

[7] Ottenjann R, Rosch W (1970). Therapie des Ulcus ventriculi mit Carbenoxolon-Natrium. Ergebnisse eines Doppelblindversuchs. Med Klin.

[8] Preedy RV, editor. Essential oils in food preservation, flavor and safety. CA, USA: Academic Press; 2015.

[9] Belinky PA, Aviram M, Fuhrman B, Rosenblat M, Vaya J (1998). The antioxidative effects of the isoflavan glabridin on endogenous constituents of LDL during its oxidation. Atherosclerosis.

[10] Wendakoon C, Calderon P, Gagnon D (2011). Evaluation of selected medicinal plants extracted in different ethanol concentrations for antibacterial activity against human pathogens. J Med Act Plants.

[11] Odey MO, Iwara IA, Udiba UU, Johnson JT, Inekwe UV, Asenye ME, Victor O (2012). Preparation of plant extracts from indigenous medicinal plants. Int J Sci Technol.

[12] Cock IE (2008). Antimicrobial activity of *Aloe barbadensis* miller leaf gel components. Int J Microbiol.

[13] Usman AK, Hazir R, Muhammad Q, Anwar H, Azizullah A, Waheed M, Zakir K, Muhammad A, Muhammad A (2015). *Alkanna tinctoria* leaves extracts: a prospective remedy against multidrug resistant human pathogenic bacteria. BMC Complement Altern Med.

[14] Valgas C, de-Souza SM, Smania EFA, Smânia A (2007). Screening methods to determine antibacterial activity of natural products. Braz J Microbiol.

[15] Steinmann D, Ganzera M (2011). Recent advances on HPLC/MS in medicinal plant analysis. J Pharm Biomed Anal.

[16] Miyasaki Y, Rabenstein JD, Rhea J, Crouch ML, Mocek UM (2013). Isolation and characterization of antimicrobial compounds in plant extracts against multidrug-resistant Acinetobacter baumannii. PLoS One.

[17] Nitalikar MM, Munde KC, Dhore BV, Shikalgar SN (2010). Studies of antibacterial activities of Glycyrrhiza glabra root extract. Int J Pharm Tech Res.

[18] Pratibha N, Sushma D, Rajinder G (2012). Screening for antioxidant and antibacterial potential of common medicinal plants in the treatment of acne. Int J Drug Dev Res.

[19] Syed F, Jahan R, Ahmed A, Khan S (2013). In vitro antimicrobial activities of *Glycyrrhiza glabra* and *Fagonia Arabica*. J Med Plant Res.

[20] Gupta A, Maheshwari DK, Khandelwa G (2013). Antibacterial activity of *Glycyrrhiza glabra* roots against certain gram-positive and gram-negative bacterial strains. J Appl Nat Sci.

[21] Makky EA, Mashitah YM, Ibrahim MM (2012). Impact of medicinal plants phytocomponents against antibiotic resistant bacteria. J Chem Pharm Res.

[22] Yancui W, Feng L, Zongsuo L, Liang P, Bangqing W, Jing Y, Yingying S, Cunde M (2017). Homoisoflavonoids and the antioxidant activity of *Ophiopogon japonicus* root. Iran J Pharm Res.

[23] Leonardo S, Alberto A, Raúl RH, Antonio AC, Cristóbal NA (2011). Ellagic acid: biological properties and biotechnological development for production processes. Afr J Biotechnol.

